# Supplementary material for: Impact of Salinity on the Energy Transfer between Pigment–Protein Complexes in Photosynthetic Apparatus, Functions of the Oxygen-Evolving Complex and Photochemical Activities of Photosystem II and Photosystem I in Two Paulownia Lines
Source: Int J Mol Sci. 2023 Feb 4;24(4):3108. doi: 10.3390/ijms24043108 (PMC9967322; doi:10.3390/ijms24043108)
Supplement: Supplementary file 1 [file ijms-24-03108-s001.zip › ijms-2161276-supplementary.pdf]

Supplementary materials

Impact of Salinity on the Energy Transfer between Pigment–Protein Complexes in Photosynthetic Apparatus, Functions of the Oxygen-Evolving Complex and Photochemical Activities of Photosystem II and Photosystem I in Two *Paulownia* Lines

Martin A. Stefanov, Georgi D. Rashkov, Ekaterina K. Yotsova, Anelia G. Dobrikova and Emilia L. Apostolova

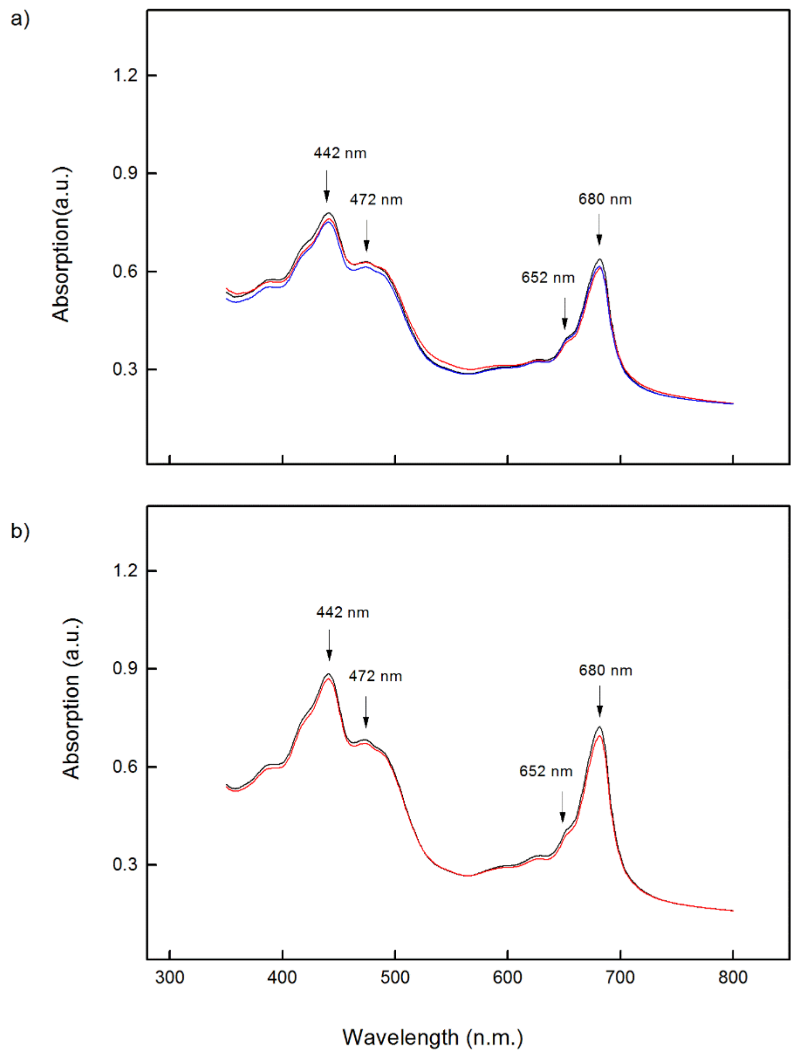

Figure S1. Absorption spectra of thylakoid membranes isolated from the leaves of *Paulownia tomentosa* x *fortunei* (TF) (a) and *Paulownia elongata* x *elongata* (EE) (b). The plants were treated for 25 days with different concentrations of NaCl, black curve (control), red curve (treatment with 100 mM NaCl) and blue curve (treatment with 150 mM NaCl).

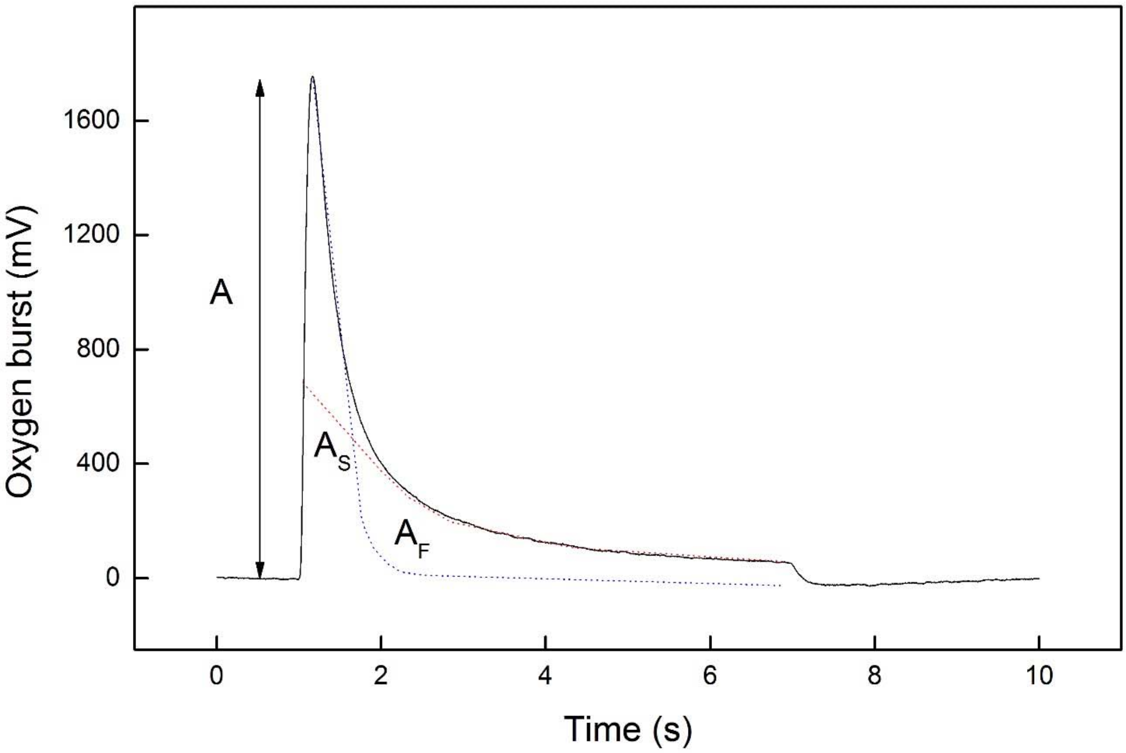

Figure S2. Initial oxygen burst kinetics under continuous irradiation of isolated thylakoid membranes from *Paulownia* leaves. The amplitude of the initial burst decay (A) and the components of oxygen burst decay: fast component (A_F) and slow component (A_S).

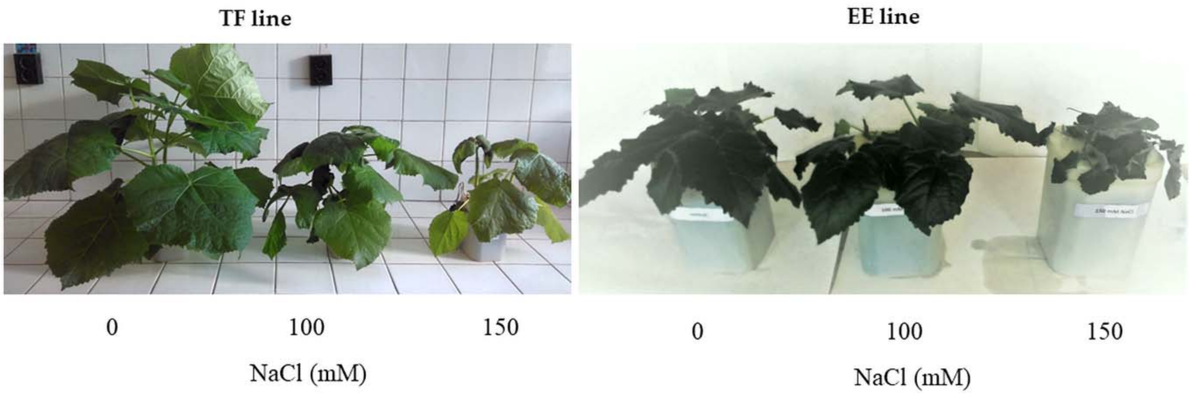

Figure S3. Effect of different NaCl concentrations on *Paulownia tomentosa* x *fortunei* (TF) and *Paulownia elongata* x *elongata* (EE).
